# Psychological and technological predictors of the physical activity intention-behavior gap: an explainable machine learning analysis

**DOI:** 10.3389/fpsyg.2025.1657506

**Published:** 2025-09-25

**Authors:** Yirong Li, Jianguang Cai

**Affiliations:** School of Physical Education, Hunan University of Science and Technology, Xiangtan, China

**Keywords:** physical activity, intention-behavior gap, double machine learning, SHAP explainable method, Health Belief Model (HBM), unified theory of acceptance and use of technology (UTAUT)

## Abstract

**Objective:**

Intention is widely regarded as the most proximal predictor of behavior. But, physical activity (PA) intentions do not invariably translate into actual exercise behavior, leaving a intention-behavior (I-B) gap. The study integrates psychological and technological frameworks to examine the mechanisms that moderate the PA I-B gap.

**Methods:**

Unlike traditional dichotomous measures of the PA I-B gap, this study employs baseline correction to derive a standardized continuous measure that quantifies the magnitude of the gap. Using survey data from 1,334 Chinese adults, we combined the Health Belief Model and the Unified Theory of Acceptance and Use of Technology within an explainable machine-learning framework to identify important predictors and their non-linear interactions.

**Results:**

The machine learning based optimal XGBoost model (R^2^ = 0.647) significantly outperforms traditional regression approaches. Perceived barriers, self efficacy, intention to use smart tools and social support emerge as the four core predictors of the PA I-B gap. Higher levels of perceived barriers and late night frequency enlarge the gap whereas greater self efficacy, perceived exercise benefits, intention to use smart tools, social support, social influence and personal innovation narrow it. The psychological cognition dimension exhibits significantly stronger predictive power than smart sports tools. These tools function primarily as auxiliary resources, and their facilitative effects differ across distinct psychological cognition levels.

**Conclusion:**

Psychological cognition and smart sports tools jointly predict the PA I-B gap. The study’s conclusions are constrained by its reliance on self-reported measures and its cross-sectional design. Future research should adopt longitudinal or experimental protocols, supplemented by objective data from wearable devices, to delineate causal pathways and illuminate the finer mechanisms underlying the gap.

## 1 Introduction

Regular physical activity (PA) is universally acknowledged as essential for enhancing health and preventing diseases. It plays a crucial role in both mitigating noncommunicable diseases such as cardiovascular conditions, cancers, and diabetes, and alleviating psychological issues like depression and anxiety. These benefits extend across various life stages, profoundly influencing overall well-being (World Health Organization, 2024). Despite a widespread intention to PA, translating these intentions into consistent PA poses a significant challenge. According to latest WHO data, 31% of adults worldwide do not achieve the recommended minimum of 150 min of moderate-intensity PA weekly (Strain et al., 2024). Despite strong PA intentions, individuals often refrain from acting on intended behaviors due to various barriers (e.g., high stress, other priorities, and waning motivation), which is a phenomenon widely recognized as the PA I-B gap (Rhodes and de Bruijn, 2013; Englert et al., 2023).

Scholars have been investigating the critical factors that influence the transition from PA intentions to actual behavior, recognizing the importance of bridging the I-B gap for enhancing PA levels and public health outcomes. Huang and Zhang (2024) empirical research on the I-B gap identified perception variables within the Health Belief Model and Theory of Planned Behavior as pivotal in converting PA intentions into tangible actions. Furthermore, the use of smart sports tools, including fitness applications (Anderberg et al., 2025) and wearable technology (Song et al., 2018), has been shown to bolster individual motivation and enhance athletic performance through monitoring and support. Healthy psychological cognition serves as an internal drive for behavior, while intelligent sports offer external support and behavioral interventions, collectively facilitating the successful translation of PA intentions into practice.

Current research predominantly employs traditional econometric methods to examine the relationships between influencing factors and the PA I-B gap. However, behavioral mechanisms frequently exhibit non-linear and high-dimensional interactive characteristics that simple parametric specifications cannot fully capture, potentially leading to biased estimates (Zhuo and Yunsong, 2025). In recent years, machine learning algorithms have become essential tools for prediction tasks in complex settings because of their capacity to handle high-dimensional covariates, capture non-linear relationships and reduce model bias. Within behavioral research, these algorithms have already been applied to learning (Su et al., 2022), internet addiction (Gan et al., 2025) and sports (Liu et al., 2023), establishing a new research paradigm. Nevertheless, machine learning prioritizes predictive accuracy, which is fundamentally different from the objective of causal inference—namely, the unbiased estimation of parameters or effects (Mullainathan and Spiess, 2017). Consequently, relying solely on predictive algorithms is insufficient for obtaining unbiased causal estimates of the relationship between independent and dependent variables.

To address this limitation, Double Machine Learning (DML) integrates high-dimensional machine learning techniques with traditional causal identification strategies by incorporating residual orthogonalization and cross-validation into a conventional causal framework. When numerous observable confounders are present, DML delivers causal effect estimates accompanied by confidence intervals. This approach demonstrates clear advantages in handling high-dimensional data, relaxing functional form assumptions, estimating conditional treatment effects, and enhancing the accuracy and reliability of causal inference (Chernozhukov et al., 2018).

Despite these strengths, complex algorithms are often perceived as black boxes, restricting the interpretability of their decision-making processes. The SHAP (SHapley Additive exPlanations) algorithm, grounded in Shapley value theory, decomposes model predictions into the marginal contributions of individual features, quantifying the influence of each variable on the outcome and presenting key pathways and threshold effects through intuitive visualizations (Lundberg and Lee, 2017).

Building on these considerations, our study first employs machine-learning algorithms to construct predictive models of the PA I-B gap and subsequently applies Double Machine Learning to conduct causal inference. SHAP is then introduced to provide transparent visual explanations. By systematically evaluating the predictive and causal impacts of psychological cognition and smart sports, this study aims to furnish rigorous evidence for the design of precise and actionable intervention strategies.

### 1.1 Physical activity intention-behavior gap

Within the domain of PA research, intention has consistently emerged as a paramount and dependable predictor, serving as a critical proximal determinant of behavioral enactment (Hagger and Chatzisarantis, 2009; Roberts et al., 2010). Nonetheless, while intentions hold significant predictive power, empirical assessments of their predictive efficacy have demonstrated that they seldom account for all variances in behavior. As highlighted in a meta-analysis by Rhodes and Dickau (2012), modest shifts in intent correspond to minimal behavioral alterations. This divergence between intended and actual behavior is termed the PA I-B gap, which constitutes the central focus of the present study.

In recent years, scholars have examined the mechanisms underlying the translation of PA intention into behavior from two complementary perspectives: the individual and the environment. At the individual level, research has primarily focused on the intrinsic properties of intention itself, including psychological attributes such as self-efficacy (Hou et al., 2022), habit strength (Di Maio et al., 2021) and personality traits (MacCann et al., 2015). Simultaneously, demographic variables including gender, age, educational attainment and health status have been demonstrated to exert differential influences on this relationship (Yang et al., 2025; Knapova et al., 2024). At the environmental level, earlier studies have shown that objective factors such as the availability and accessibility of sports facilities (Santinha et al., 2022), together with subjective factors such as social support (Schumacher et al., 2021) and cross-cultural variations between individualistic and collectivistic exercise cultures (Gurleyik et al., 2022), have moderated the I-B pathway. Collectively, these elements have formed a complex, intertwined network of potential confounders.

Despite this progress, extant research has predominantly relied on traditional statistical techniques, namely linear regression (Schumacher et al., 2021) and mediation or moderation models (Hou et al., 2022; Yang et al., 2025; Knapova et al., 2024). Although these approaches have established several significant associations, they have remained limited in their capacity to identify critical variables precisely within high-dimensional feature spaces and to characterize nonlinear relationships adequately. Moreover, minor alterations in model specifications have yielded divergent interpretations (Rebar et al., 2019), thereby undermining the robustness and generalisability of the findings.

Much of the existing research employs a categorical framework to measure the I-B gap, categorizing individuals into quadrants based on their intentions (intentioned/non-intentioned) and outcomes (successful/unsuccessful)(Sheeran, 2002). Additionally, some studies have evaluated the correlation between intention strength and behavior through fixed statements, such as I plan to engage in PA at least N times per week, coupled with response scales that have gauged the degree of agreement (Maltagliati et al., 2025). Although these methods have been prevalent in PA research, they have exhibited inherent limitations: they have failed to provide quantitative analyses of the gap’s magnitude and have struggled to capture individual nuances. For instance, existing measurement techniques have been unable to differentiate accurately and quantify the gap between individuals who have aimed for seven weekly PA sessions but have achieved only five and those who have intended to exercise once but have failed to do so (Burnett et al., 2018). Consequently, comparing I–B gaps across individuals with varying baseline levels has remained challenging, thereby constraining the precise assessment of the gap’s extent.

### 1.2 HBM-UTAUT theoretical model

This study centrally aims to investigate the discrepancy between individuals’ PA intentions and their actual behaviors. The majority of prior research has concentrated on intra-individual factors, including personality differences (Maltagliati et al., 2025), PA procrastination (Miao et al., 2024) and PA preferences (Rebar et al., 2016), often neglecting the impact of external environmental factors on the I-B relationship. Amidst the rising prevalence of digital lifestyles, the significance of smart sports tools as convenient and efficient instruments for enhancing PA intentions and PA has garnered increasing attention (Yang and Koenigstorfer, 2021). By integrating the Health Belief Model with the Unified Theory of Acceptance and Use of Technology (UTAUT), this study establishes a comprehensive theoretical framework that encapsulates both psychological cognition and technological support. This psycho-technology dual-path model offers a holistic view to elucidate the disparities between PA intentions and behaviors, thereby furnishing a theoretical foundation for devising effective intervention strategies.

The Health Belief Model (HBM) encompasses components such as perceived benefits, perceived barriers, perceived severity and self-efficacy, which are pivotal in assessing attitudes toward health conditions (Rahmati-Najarkolaei et al., 2015). It stands as one of the seminal theories in behavioral health. This model, renowned for its ability to identify, elucidate, and forecast health-related behaviors, as well as to guide preventive measures, has been extensively applied to anticipate and interpret the motivational factors driving PA across diverse demographics, including the elderly (Qiao et al., 2021), pregnant women (Shafieian and Kazemi, 2017) and college students (Sheng et al., 2023). Given its relevance to the study of individual PA, and considering its frequent application in examining the PA I-B gap, the HBM is integrated into the theoretical framework of the present research.

The Unified Theory of Acceptance and Use of Technology (UTAUT), introduced by Venkatesh et al. (2003), offers a comprehensive framework that synthesizes insights from eight pre-existing models. This theory primarily utilizes four core constructs—performance expectations, effort expectancy, social influence and facilitating conditions—to elucidate users’ intentions and behaviors regarding the adoption of specific technologies, alongside other significant factors. Smart sports refers to the use of smart devices, apps, virtual reality and other technical means to provide individuals with PA guidance, feedback and motivation. Studies have shown that UTAUT is a robust predictive model of technology acceptance, and it is widely used to study smart sports initiatives, including live sports video platforms (Xiang et al., 2024), fitness applications (Liu et al., 2019), and smart wearables (Seol et al., 2017). The UTAUT model goes beyond the binary analysis of technology use to delve into how various attributes of intelligent motion affect the motor I-B gap (Wei et al., 2021) and proves particularly good at assessing users’ propensity to accept new technologies. Therefore, this study integrates UTAUT into its theoretical framework to enhance the understanding of the adoption of intelligent motor technology and its impact on motor behavior.

### 1.3 Double machine learning

Over the past decade, machine-learning algorithms have been widely adopted by social scientists for data generation and prediction tasks. Across economics, sociology and psychology, causal identification has become the central concern of empirical inquiry (Abadie and Cattaneo, 2018). For machine-learning algorithms engaged in data prediction, it is sufficient to establish correlations among variables; causal relationships are not required. Yet Kleinberg points out that the principal goal of most machine-learning models is prediction: observable correlations between features and outcomes suffice, and causal structure is dispensable (Kleinberg et al., 2015). This orientation leads many algorithms to neglect the underlying causal chain, focusing solely on predictive performance and thereby creating a methodological gap with the mainstream causal-identification literature in the social sciences. Nevertheless, machine learning and causal inference are not inherently incompatible. Chernozhukov et al. (2018) integrate the high-dimensional flexibility of machine learning with the identification strategies of classical causal inference to propose Double Machine Learning (DML). By retaining the adaptability of machine-learning algorithms while leveraging residual orthogonalization and cross-validation, DML delivers unbiased estimates of causal effects and offers a novel and important technical pathway for causal identification in the social sciences.

DML is implemented in two stages. The first stage is a pure prediction task aimed at obtaining highly accurate forecasts of the outcome variable. Guided by variable types and data structure, researchers use cross-validation to select the best performing model from among SVM, Random Forest and XGBoost, and extract residuals for subsequent orthogonalization. The second stage estimates the causal effect of the treatment variable on the outcome. Given the potential non-linear relationships among variables, polynomial regression or non-parametric strategies such as causal random forests are typically employed. Non-parametric approaches avoid prespecified functional forms and allow confidence intervals to be constructed through bootstrap sampling, with traditional hypothesis tests or interval estimates providing the statistical basis for inference (Chernozhukov et al., 2018). It should be noted, however, that DML is not without limitations; in particular, algorithmic opacity and insufficiently revealed parameter heterogeneity remain concerns. Consequently, after obtaining causal-effect estimates, one can incorporate the SHAP explainable machine-learning framework to decompose model predictions into the marginal contributions of individual features and to conduct case-level heterogeneity analyses.

## 2 Methods

### 2.1 Data source

To ensure the representativeness and validity of the smart-sport dimension data, the sampling framework was aligned with the gender and age distribution of habitual smart-sport users. Under the constraint of an approximately balanced gender ratio, the target population was restricted to adults aged 18–45 years. Accordingly, questionnaires were distributed via Wenjuanxing to residents in Changsha, Zhuzhou and Xiangtan, Hunan Province, between 1 and 20 April 2025. The study protocol and online informed consent form were approved by the Ethics Committee of Hunan University of Science and Technology, and all participants gave written informed consent before they began the questionnaire. A total of 1,428 questionnaires were received; after excluding questionnaires completed in <5 min or >10 min and those with clearly consistent answers, 1,334 valid questionnaires were retained. The final sample comprised 49.1% males and 50.9% females; 86.9% were aged 18–45. Educational attainment ranged from below junior high to master’s degree, with 87.9% having completed senior high school or higher. The two most common occupational categories were private-sector employees (27.8%) and students (27.3%). The sample’s demographic composition closely mirrors the actual user profile of smart-sports technologies, indicating strong representativeness for the population of interest.

### 2.2 Variable description

#### 2.2.1 Physical activity intention-behavior gap (PA I-B gap)

Based on the Physical Activity Rating Scale-3 (PARS-3) (Liang, 1994), operationalisation proceeded in two steps. First, each participant’s actual PA score was calculated as intensity × duration × frequency (maximum = 100). Second, under unconstrained conditions, each participant’s ideal PA score was computed as ideal intensity × ideal duration × ideal frequency (maximum = 100). Because baseline PA levels differ markedly between individuals, a simple raw difference can misrepresent the true gap: for example, an absolute discrepancy of 20 points is less consequential for a high-achiever (actual = 60, ideal = 80) than for a low-achiever (actual = 20, ideal = 40). Following standard practice in medical research, we applied baseline correction to convert the absolute difference into a relative index, ΔZ = (Ideal − Actual)/σ_actual, where σ_actual denotes the standard deviation of the actual scores across the entire sample. This index quantifies how many standard deviations an individual’s I-B gap deviates from the population mean gap, while preserving the validity of PARS-3 and eliminating baseline heterogeneity. The formula as follows:


$$\mathrm{\Delta Z}=\frac{\text{Ideal}-\text{Actual}}{\sigma \_\text{actual}}.$$

To evaluate the utility of ΔZ, a machine-learning model with ten-fold cross-validation was used to compare its predictive performance with that of the traditional absolute gap (Z). ΔZ achieved R^2^ = 0.647, significantly outperforming Z (R^2^ = 0.47), thereby demonstrating superior statistical performance and interpretability and establishing ΔZ as a robust, comparable core variable for subsequent analyses.

#### 2.2.2 Health Belief Model (HBM) and the Unified Theory of Acceptance and Use of Technology (UTAUT)

Drawing on the PA health belief scale designed by Huang and Zhang (2024) and Dai et al. (2011), this study assessed individual HBM across five dimensions: perceived benefits (BEN), affective attitude (AT), perceived barriers (BAR), perceived severity (SEV), and self-efficacy (SE). Smart sports tools refer to intelligent products that enhance the athletic experience through the application of smart technology, which primarily include fitness apps, smart wearable devices and exercise-focused short videos. Building on the UTAUT (Unified Theory of Acceptance and Use of Technology) scale designs by Venkatesh et al. (2003) and Featherman and Pavlou (2003), this study measures respondents’ perceptions of smart sports tools across seven dimensions: performance expectancy (PE), effort expectancy (EE), social influence (SI), facilitating conditions (FC), personal innovation (PN), perceived risk (PR), social support(SS) and usage intention (UI). All items were evaluated using a 7-point Likert scale.

Reliability analysis revealed that the Cronbach’s alpha coefficients for all latent variables ranged from 0.72 to 0.89, exceeding the threshold of 0.70, indicating strong internal consistency of the scales. Exploratory factor analysis demonstrated that the KMO values for the HBM and UTAUT scales were 0.848 and 0.786, respectively (both above 0.60), and Bartlett’s test of sphericity yielded a significance level of *p* < 0.001, confirming the structural validity of the questionnaire for factor analysis. Confirmatory factor analysis indicated that the composite reliability (CR) for each factor was greater than 0.7, and the average variance extracted (AVE) was greater than 0.5, suggesting that all dimensions in this study have good construct reliability and convergent validity, aligning with theoretical expectations. The operationalization of the items for each variable and their validity assessment results are presented in Table 1. It should be noted that the standardized factor loading for BAR1 (The area where I reside lacks suitable sports facilities) is at the threshold level (loading = 0.504), yet it still exceeds the customary lower bound of 0.50. As a core measure of objective environmental accessibility, this item has been empirically demonstrated in the extant literature to significantly facilitate PA behavior by modulating psychological states (Xue and Li, 2023). Balancing empirical evidence with theoretical relevance, we retained BAR1 to enhance the model’s coverage of contextual factors.

**Table 1:** Operationalized items and validity test results.

Variables	Latent variables	Operationalize the item	Standard load factor	AVE	CR
HBM					
BEN	BEN1	PA can promote health	0.850	0.778	0.875
	BEN2	PA can prevent or control chronic diseases	0.913		
AT	AT1	Moderate PA level is very enjoyable for me.	0.897	0.742	0.851
	AT2	Moderate PA level is very easy for me	0.824		
BAR	BAR1	The area where I reside lacks suitable sports facilities	0.504	0.546	0.797
	BAR2	I have not yet found a suitable form of exercise	0.728		
	BAR3	I find exercise is too exhausting, too painful and lacks joy	0.730		
	BAR4	I do not have much time and energy to engage in regular exercise	0.741		
	BAR5	I find it difficult to maintain exercise due to a lack of companions	0.651		
SEV	SEV1	Lack of PA can make me feel tired and listless	0.723	0.613	0.825
	SEV2	Lack of PA can increase my risk of chronic disease	0.761		
	SEV3	Lack of PA can make me susceptible to anxiety or depression	0.858		
SE	SE1	I believe that I will be able to learn new PA content	0.829	0.787	0.936
	SE2	Even if I encounter difficulties in PA, I believe I can do it	0.934		
	SE3	I believe I can overcome various difficulties in pursuing PA	0.942		
	SE4	I believe I can complete a pre-made PA plan	0.837		
UTAUT					
PE	PE1	Smart sports tools are very helpful to me	0.915	0.816	0.947
	PE2	Smart sports tools improve exercise efficiency	0.938		
	PE3	Smart sports tools enhance sports knowledge and skills	0.919		
	PE4	Smart sports tools aid in long-term exercise adherence	0.844		
EE	EE1	Using smart sports tools is very easy for me	0.900	0.832	0.939
	EE2	Smart sports tools are powerful and user-friendly	0.915		
	EE3	I can conveniently use smart sports tools for training support	0.921		
SI	SI1	People around me use smart sports tools	0.836	0.798	0.922
	SI2	Important people in my life use smart sports tools	0.955		
	SI3	Important people in my life recommend me use smart sports tools	0.884		
FC	FC1	I have the smart sports hardware, like apps and fitness trackers	0.820	0.706	0.878
	FC2	I have the environment needed for smart sports tools	0.871		
	FC3	I can solve exercise issues through smart sports tools	0.828		
PN	PN1	Smart sports tools recommend plans based on my preferences‌	0.867	0.789	0.937
	PN2	Smart sports tools customize training intensity based on my fitness level‌	0.864		
	PN3	Smart sports tools offer flexible workout plans and locations	0.925		
	PN4	Smart sports tools provide multiple options for my choice	0.895		
PR	PR1	I am worried that smart sports tools may leaking my data	0.663	0.606	0.883
	PR2	I am worried that smart recommendations will not meet my needs	0.819		
	PR3	I am worried that paid memberships or courses will not achieve the desired results	0.858		
	PR4	I am worried that I must buy specific gear to use core smart features‌	0.746		
	PR5	I am worried that virtual guidance may not be as effective as instruction from a real coach.	0.822		
SS	SS1	I can find like-minded exercise partners through smart sports tools	0.737	0.679	0.894
	SS2	I can discuss data generated by smart sports tools with my friends.	0.814		
	SS3	I feel supported and encouraged through smart sports tools	0.864		
	SS4	I gain social recognition through smart sports tools	0.875		
UI	UI1	I plan to keep using smart sports tools in the future	0.929	0.844	0.956
	UI2	I am willing to recommend smart sports tools to others	0.936		
	UI3	I intend to continue using smart sports tools	0.883		
	UI4	I will maintain or increase use of smart sports tools	0.927		

#### 2.2.3 Characteristics of PA

Research indicates that PA I-B gap is shaped by a multifaceted profile of PA engagement, including: (1) the proficiency level in PA, which indicates an individual’s experience and skill in PA (Gao et al., 2021) and is a pivotal predictor of PA behavioral intentions; (2) modes of exercise engagement, which cover various participation formats such as individual workouts, partnered activities, and club-based sessions (Gut et al., 2020), influencing the frequency and consistency of PA; (3) exercise motivations, reflecting the range of reasons individuals have for engaging in sports, including health, leisure, socializing, weight management, and personal interests (Liu et al., 2023), with different motivations correlating with the vigor and persistence of PA; and (4) the economic investment in sports, which pertains to the financial outlay on sports gear and gym subscriptions (Chen et al., 2024). These dimensions collectively contribute to understanding and potentially narrowing the I-B gap in PA.

#### 2.2.4 Basic demographic information

This study selects demographic and health-related behavioral variables to control for potential confounding factors. Demographic variables include gender, age, education level, current occupation and monthly income level; health-related characteristics primarily consist of perceived health status, frequency of staying up late, and Body Mass Index (BMI), all of which are incorporated into the statistical analysis. The assignment of values to each variable and descriptive statistical analysis are presented in Table 2.

**Table 2:** Variables description and descriptive statistical analysis.

Variables	Variable description	M	SD
PA_level	PA Level: 1 = No experience; 2 = Beginner; 3 = Intermediate; 4 = Advanced	2.27	0.777
PA_organize	PA Format: 1 = Alone; 2 = With a partner; 3 = Club or group	1.36	0.479
PA_purpose	Number of PA Motivations (including strengthening, stress relief, interest, skill learning, socializing, willpower and fitness): 1 to 7	4.11	1.856
PA_comsume	PA Expenditure (including sportswear, equipment, venue rental and membership fees, yuan/month): 1 = 0; 2 = 1, 100; 3 = 101, 300; 4 = 301, 500; 5 = Over 501	1.82	0.980
Sm_duration	Smart tool usage duration: 0 = Non-user; 1 = 1–3 months; 2 = 3–6 months; 3 = 6–12 months; 4 = 1–2 years; 5 = Over 2 years	1.45	1.324
Sm_frequency	Smart tool usage frequency: 0 = Non-user; 1 = ≤3 times/month; 2 = 1–2 times/week; 3 = 3–4 times/week; 4 = ≥5 times/week	1.57	1.109
Sm_number	Number of Smart Tools Used (including Apps, video and wearables): 1–3	1.51	1.152
Sm_purpose	Number of smart-tool use purposes (including planning, guidance, knowledge, data, gear, community and wellness):1–7	2.93	2.017
Sex	Gender: 1 = Male; 2 = Female	1.51	0.500
Age	Age: 1 = 18–25; 2 = 26–35; 3 = 36–45; 4 = Over 45	2.13	1.046
Career	Occupation: 1 = Government/Public sector; 2 = Private Sector; 3 = Freelancer; 4 = Student	2.57	1.104
Education	Education Level: 1 = Junior High or below; 2 = High School; 3 = Bachelor’s; 4 = Master’s or above	2.58	0.874
Consumption_level	Disposable income per month (yuan): 1 = Up to 1,500; 2 = 1,500–3,000; 3 = 3,001–5,000; 4 = 5,001–8,000; 5 = Over 8,001	2.61	0.841
Health	Health Status: 1 = Very unhealthy; 2 = Unhealthy; 3 = Average; 4 = Healthy; 5 = Very Healthy	3.48	0.910
Midnight	Frequency of staying up late (after midnight): 1 = Never; 2 = ≤3 times/month; 3 = 1–2 times/week; 4 = 3–5 times/week; 5 = Almost every day	3.44	1.205
BMI	$\frac{\mathrm{KG}}{M^{2}}$	21.31	2.948

Based on the questionnaire design, 68 sub-variables were systematically encoded and integrated into 30 composite variables, consisting of one outcome variable and 29 predictor variables, with no missing observations. Among the predictors, continuous variables were standardized to a mean of zero and a standard deviation of one via the z-score method, whereas categorical variables were recoded using one-hot encoding. Subsequent Pearson correlation analyses revealed that all predictors were<0.60, confirming independence and absence of multicollinearity, which allowed the variables to be entered into the subsequent modelling procedures.

### 2.3 Double machine learning construction

Compared with traditional regression, Double Machine Learning (DML) offers clear advantages in handling high-dimensional covariates, multicollinearity and nonlinear relationships. Its core logic is a two-step procedure: first, a high-precision predictive model is constructed; second, causal effects are estimated using parametric or non-parametric strategies. Guided by this paradigm, the present study established the following analytical pipeline.

#### 2.3.1 Construction of a high-precision predictive model

To quantify the PA I-B gap, we systematically evaluated the explanatory power of five algorithms—ordinary least squares (OLS), decision tree (D-Tree), support vector machine (SVM), random forest (R-Forest) and extreme gradient boosting (XGBoost)—against a high-dimensional, nonlinear data structure. The workflow comprised three sequential stages: training, validation, and hyper-parameter optimisation. After an 80:20 random split of the data into training and testing sets, a five-fold cross-validated grid search was performed on the training data. Model performance was adjudicated using mean squared error (MSE), root-mean-square error (RMSE), mean absolute error (MAE), and the coefficient of determination (R^2^).

Table 3 summarized the comparative results. XGBoost achieved the lowest MSE, RMSE, and MAE, while simultaneously registering the highest R^2^(0.613), thereby outperforming the alternative algorithms by a clear margin. Accordingly, the grid-optimized XGBoost model was selected as the base learner for the subsequent causal-inference pipeline.

**Table 3:** Comparison of the performance of machine learning algorithms.

Performance metrics	OLS	SVM	D-Tree	R-Forest	XG Boost
MSE	0.182	0.165	0.295	0.140	0.101
RMSE	0.427	0.407	0.543	0.374	0.318
MAE	0.336	0.295	0.371	0.281	0.233
R^2^	0.396	0.460	0.128	0.535	0.613

#### 2.3.2 Causal effect estimation

Upon validating the superior predictive performance of the XGBoost model, we incorporated it into the ‌Causal Forest DML‌ framework to estimate the ‌Average Treatment Effect (ATE)‌ of key features on the ‌PA I-B gap‌. In this framework, the continuous outcome variable Y represents the PA I-B gap, the treatment variable T consists of the core features selected via XGBoost-SHAP importance analysis, and the confounding variables W include all observed covariates except the specific treatment variable under investigation. To eliminate the influence of confounding variables and ensure unbiased estimation of the causal effect *θ* of T on Y, the estimation process strictly follows the canonical two-stage procedure of DML, incorporating residual orthogonalization and cross-fitting techniques.

1. Cross-Fitting and Residual Estimation. By leveraging the XGBoost model to assess the impact of the confounding variable W on the dependent variable Y and the treatment variable T, the estimated values ĝ(W) and ĥ(W) are obtained, respectively. Subsequently, the residuals E_y_ and E_t_ of the two predictive models are computed, with the specific formulas as follows:

$$E_{y}=Y-\hat{g}(W),\hat{g}(W)=E(Y|W)$$

$$E_{t}=Y-\hat{h}(W),\hat{h}(W)=E(Y|W)$$

2. ATE Estimation. The residual E_t_ is employed to fit E_y_, aiming to estimate the parameter θ, which serves as an unbiased estimator of the ATE between T and Y:‌

$$E_{y}={\theta_{0}}^{*}E_{t}+\in$$

3. Mitigation of Overfitting Bias through Cross-Fitting. To ensure the robustness of the estimation, the K-Fold cross-validation method is adopted, where the data are partitioned into K folds, and the estimation process is repeated K times. This separation of model training and residual calculation effectively controls the systematic errors in the residuals. The final estimate of the causal effect is given by:

$$\theta =\frac{1}{K}\sum_{1}^{k}\theta_{k}$$

In brief, the core procedure of DML consists of two stages: first, XGBoost is employed to model both the outcome variable Y and the treatment variable T, and the residuals of Y and T are obtained by subtracting the predicted values from the observed values; second, a non-parametric model is used to fit the relationship between these residuals, thereby estimating the causal effect of the key feature on Y. Repeating the above steps via K-fold cross-fitting effectively controls overfitting bias and yields a robust and interpretable estimate of the ATE along with its confidence interval.

## 3 Results

### 3.1 Construction of the optimal model

To ensure the robustness of the model, this study employed a method of multiple random splits to mitigate the stochastic effects of data partitioning. Specifically, the sample was divided using 20 distinct random seeds for proportional stratification, followed by a grid search with 10-fold cross-validation via GridSearchCV to ascertain the optimal hyperparameters. Through this process, the XGBoost model achieved a mean coefficient of determination (R^2^) of 0.613, which was significantly higher than that of other machine learning algorithms, reaffirming its status as the superior predictive model. With the optimal hyperparameter configuration, the R^2^ of the XGBoost model further increased to 0.647, indicating its high accuracy and generalizability in forecasting the PA I-B gap among college students. The optimal model hyperparameters are detailed in Table 4.

**Table 4:** Parameter settings of the XGBoost algorithm.

Model parameters	Meaning of the parameter	Parameter results
N_estimators	Number of iterations	300
Max_depth	Depth of trees	5
Learning_rate	Learning rate	0.05
Subsample	Proportion of samples randomly selected per iteration	0.8
Colsample_bytree	Proportion of features randomly sampled for each tree	0.9
Reg_lambda	L2 regularization term on weights.	3.0
Reg_alpha	L1 regularization term on weights.	0.1
Min_child_weight	Minimum sum of instance weight needed in a child node.	3
Gamma	Minimum loss reduction required to make a further partition.	0.01

### 3.2 Analysis of important features

**Figure 1:**
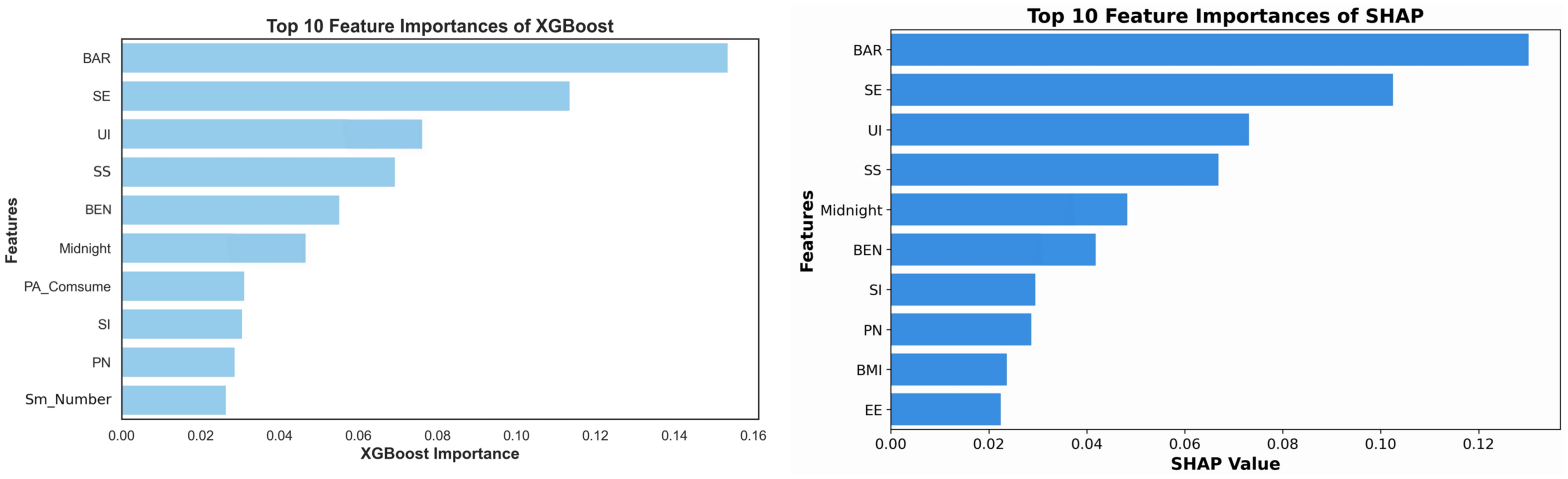
Ranking of feature importance.

Accurately identifying and ranking key predictors is a central task in machine learning forecasting. Using the optimal XGBoost algorithm and the SHAP framework, this study independently assessed the relative importance of features contributing to the PA I-B gap. Figure 1 presents the ten most influential variables obtained from each method; eight of the ten highest-ranked features overlap between algorithms, attesting to model stability and the persistent salience of these predictors. Although their exact ranks differ slightly, perceived barriers (BAR), self-efficacy (SE), intention to use smart tools (UI) and social support (SS) consistently occupy the top four positions and jointly account for more than 50% of the total SHAP value. Additionally, aggregating the SHAP contributions of these four variables reveals that the psychological-cognition dimension (BAR+SE) accumulates to 0.233, approximately 1.7 times the contribution of the smart-sport dimension (UI + SS = 0.140). Thus, compared with technological factors, psychological cognition exerts a substantially stronger influence on attenuating the gap, whereas smart-sport tools play a secondary, supportive role. Moreover, the frequency of staying up late, as an indicator of an unhealthy lifestyle, maintains a prominent rank with a stable SHAP value of approximately 0.05, further corroborating the significant predictive value of sleep behavior for the gap.

### 3.3 Explanatory analysis of important features

**Figure 2:**
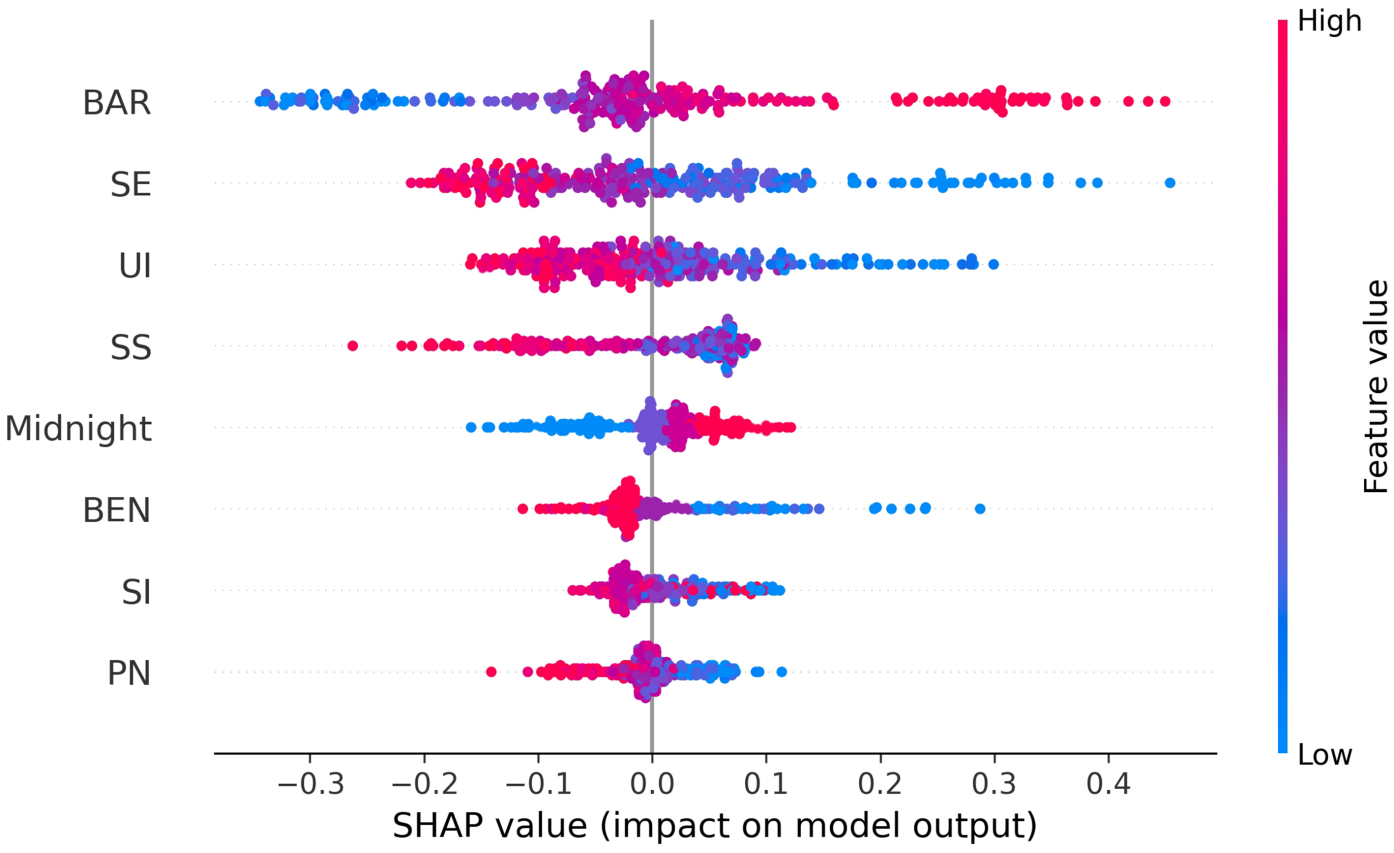
SHAP beeswarm plot of important features.

Drawing on eight overlapping key features, this study employed the SHAP framework to decompose the average marginal contribution of each predictor to the XGBoost model’s output and to clarify its directional effect. Figure 2 presents a SHAP summary plot in which features are arranged in descending order of importance along the vertical axis; each dot represents an individual sample, its horizontal position indicating the marginal effect on the predicted outcome, while the blue-to-red color gradient denotes the corresponding feature value from low to high.

The results indicate that BAR constitute the foremost predictor, exerting a positive effect whereby higher values significantly widen the PA I-B gap. SE ranks second, with elevated values markedly narrowing the gap. Subsequently, UI and SS, key dimensions of smart sports technology, demonstrate negative effects, such that higher values are associated with a smaller gap. Late-night behavior exhibits a upward association, with increasing frequency corresponding to an enlarged gap. In contrast, BEN, SI and PN, although negatively related to the gap, display SHAP absolute values significantly lower than those of the top four predictors, indicating a relatively limited influence. Overall, psychological-cognitive variables exert a greater contribution to the I-B gap than smart-sports variables, and the directional effects are fully consistent with theoretical expectations.

### 3.4 Causal analysis of important features

After partialling out high-dimensional confounders, we estimated the Average Treatment Effects (ATEs) of eight focal variables on the PA I-B gap using Causal Forests. All estimates were obtained within a Double Machine Learning framework that combined residual orthogonalization with cross-fitting; standard errors and 95% confidence intervals (CI) were constructed via 5,000 bootstrap replications using normal approximation.

Table 5 shows that the ATEs for BAR and Midnight are significantly upward [BAR: 0.186, 95% CI = (0.160, 0.211), *p* < 0.001; Midnight: 0.108, 95% CI = (0.088, 0.129), *p* < 0.001]. A one-standard-deviation increase in either variable widens the PA I-B gap by 0.186 and 0.108 units, respectively. SE, UI, BEN, SS, SI and PN yield significantly downward ATEs. Among them, SE exhibits the largest effect size [−0.157, 95% CI = (−0.192, −0.122), *p* < 0.001], indicating the strongest causal impact on narrowing the gap. None of the 95% CIs include zero, reinforcing the robustness of these causal estimates.

**Table 5:** ATE and 95% CI of important variables.

Variables	ATE	Standard error	95% CI
BAR	0.186^***^	0.013	[0.160, 0.211]
SE	−0.157^***^	0.018	[−0.192, −0.122]
UI	−0.118^***^	0.015	[−0.148, −0.088]
SS	−0.076^***^	0.011	[−0.098, −0.054]
BEN	−0.116^***^	0.010	[−0.136, −0.096]
Midnight	0.108^***^	0.011	[0.088, 0.129]
PN	−0.095^***^	0.016	[−0.126, −0.0646]
SI	−0.026^***^	0.009	[−0.044, −0.008]

Number = 1,334; R^2^ = 0.647; *p < 0.01; **p < 0.05; ***p < 0.001.

### 3.5 Dependency explanation of single-variable

Under the established causal significance, we generated SHAP dependence plots with the feature value on the horizontal axis and the SHAP value on the vertical axis; values above zero indicate an amplification of the PA I-B gap, whereas values below zero indicate a reduction. A LOWESS curve was superimposed to delineate the linear and non-linear associations with the gap.

**Figure 3:**
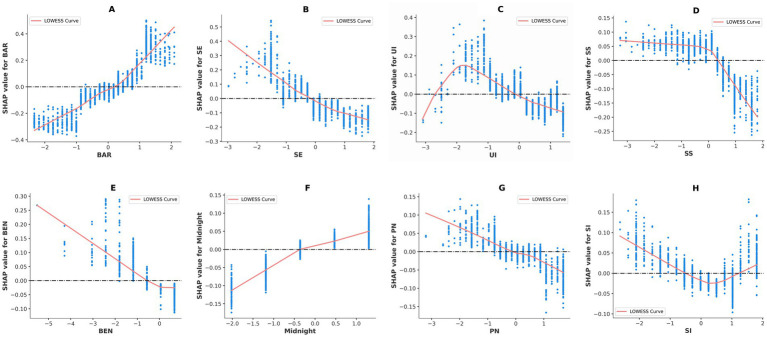
SHAP dependence plot of important features.

Figure 3 shows that BAR, SE, BEN, Midnight, and PN follow smooth monotonic trajectories without inflection points, consistent with linear relationships. As BAR and Midnight increase, their positive contributions to SHAP values rise linearly, thereby widening the I-B gap; conversely, higher levels of SE, BEN, and PN linearly attenuate the gap. UI, SS, and SI exhibit non-linear patterns. UI displays an inverted U-shaped relationship: when the UI index falls below −2.5 or exceeds 0, SHAP values drop to low levels, suggesting that both minimal and intensive use of smart-sport tools can markedly reduce the gap. SS exhibits a clear threshold effect: SHAP values decline sharply once the SS index surpasses 0.5, indicating that high social support significantly shortens the gap. SI presents a mild U-shaped curve: SHAP values are lowest when the SI index lies between 0.4 and 1.2, implying that either insufficient or excessive social influence may enlarge the gap. Collectively, the coexistence of linear and non-linear patterns reveals a multidimensional and complex mechanism underlying the formation of the PA I-B gap.

### 3.6 Heterogeneity analysis

Basing on the combined SHAP evidence, the psychological-cognition dimension exerts a significantly larger aggregate effect than the smart-sports-tools dimension, confirming its dominant role in narrowing the PA I-B gap, whereas the latter serves primarily an auxiliary function. To examine whether this dominan-auxiliary relationship varies across psychological-cognition levels, we dichotomised the sample at the median values of BAR and SE and estimated heterogeneous treatment effects using DML framework. The smallest subgroup (*n* = 611) exceeds the 250–500 cases threshold recommended for Causal-Forest subgroup analyses (Bonander and Svensson, 2021), ensuring adequate statistical power.

Table 6 reveals a consistent pattern of high-cognition activation yet low-cognition inhibition among the smart-sports-tool dimensions. Specifically, three findings emerge: (1) UI, SS, and PN constitute the core tool effects. All three variables significantly promote the translation of intention to behavior (*p* < 0.001; 95% CIs exclude zero). The effects are strongest when self-efficacy is low: PN = –0.100, SS = –0.100 and UI = –0.094. (2)Multi-dimensional activation among cognitively advantaged groups. In the low-BAR and high-SE subgroups, PE and EE exhibit significant positive effects (*p* < 0.05; 95%CIs exclude zero). Additionally, PR is significant only in the low-BAR group (ATE = 0.054, *p* < 0.05) and SI is significant only in the high-SE group (ATE = -0.039, *p* < 0.001). Overall, individuals with higher psychological-cognition levels are more able to leverage the diverse facilitative attributes of smart-sports tools. (3)Restricted tool functionality among cognitively disadvantaged groups. Beyond UI, SS, and PN, only FC is significant in the low-SE subgroup (ATE = −0.027, *p* < 0.05); all other dimensions carry confidence intervals that span zero. Thus, when psychological-cognition levels are low, the facilitative potential of smart-sports tools is markedly constrained.

**Table 6:** Heterogeneity analysis by subgroups.

Variables	Lower BAR		Higher BAR		Lower SE		Higher SE	
	ATE	95% CI	ATE	95% CI	ATE	95% CI	ATE	95% CI
PE	−0.030^**^ (0.016)	[−0.062, −0.001]	0.022 (0.021)	[−0.019, 0.062]	0.003 (0.014)	[−0.025, 0.031]	−0.046^***^ (0.013)	[−0.071, −0.021]
EE	−0.039^***^ (0.015)	[−0.069, −0.010]	0.010 (0.015)	[−0.019, 0.039]	0.031 (0.023)	[−0.014, 0.076]	−0.029^**^ (0.013)	[−0.055, −0.008]
SI	−0.017 (0.020)	[−0.056, 0.023]	−0.019 (0.011)	[−0.042, 0.003]	−0.006 (0.021)	[−0.048, 0.035]	−0.039^***^ (0.016)	[−0.070, −0.010]
FC	−0.005 (0.019)	[−0.033, 0.043]	−0.023 (0.014)	[−0.050, 0.004]	−0.027^**^ (0.014)	[−0.054, 0.000]	−0.021 (0.020)	[−0.060, 0.018]
PN	−0.081^***^ (0.022)	[−0.124, −0.039]	−0.055^***^ (0.020)	[−0.095, −0.014]	−0.100^***^ (0.020)	[−0.139, −0.060]	−0.069^***^ (0.017)	[−0.102, −0.035]
PR	0.054^**^ (0.025)	[0.005, 0.102]	−0.018 (0.013)	[−0.043, 0.008]	0.011 (0.024)	[−0.036, 0.059]	0.003 (0.023)	[−0.042, 0.048]
SS	−0.077^***^ (0.013)	[−0.102, −0.052]	−0.062^***^ (0.014)	[−0.089, −0.035]	−0.100^***^ (0.016)	[−0.131, −0.069]	−0.056^***^ (0.020)	[−0.096, −0.016]
UI	−0.108^***^ (0.023)	[−0.153, −0.064]	−0.051^**^ (0.023)	[−0.096, −0.006]	−0.094^***^ (0.023)	[−0.139, −0.050]	−0.068^***^ (0.017)	[−0.101, −0.035]
Number	611		733		715		629	
R^2^	0.532		0.526		0.565		0.578	

*p < 0.01; **p < 0.05; ***p < 0.001, standard error in ().

## 4 Discussion

The study analyzed 1,344 valid Chinese questionnaires, employing five machine learning algorithms (‌OLS, SVM, DT, RF and XGBoost‌) with hyperparameter optimization to construct the best-fitting prediction model for the ‌PA I-B gap‌. Building upon the optimal model, we estimated the ‌average treatment effect‌ of important predictors on the PA I-B gap by employing ‌residual orthogonalization and cross-validation‌ to account for high-dimensional observable confounders. ‌SHAP-based visualization‌ further elucidated the mechanisms underlying these effects. Compared to conventional regression methods, the ‌interpretable DML framework‌ significantly enhanced both predictive accuracy and causal interpretability, thereby establishing a novel methodological paradigm for PA behavior research.

Our findings indicate that perceived barriers, self-efficacy, intention to use smart tools and social support are the primary determinants of the PA I-B gap. These results provide empirical evidence for the synergistic role of psychological health cognition and smart sports support in moderating the gap. Unlike previous studies that have focused on either psychological cognition (Burnett et al., 2018; Miao et al., 2024) or smart sports (Wang and Dai, 2022) in isolation, our study emphasizes the combined effect of both factors. Among the top four predictors, the aggregated SHAP value of the psychological-cognition dimension (0.233) was approximately 1.7 times that of the smart-sports-tools dimension (0.140), indicating that the former exerts dominant predictive power over the PA I-B gap.

The Health Belief Model proposes that perceived barriers such as lack of energy or interest reduce exercise motivation (Wu et al., 2020). Smart sports tools can mitigate these barriers and reinforce exercise adherence through gamification and social interaction (Laranjo et al., 2021), yet their effectiveness hinges on users’ ability to overcome intrinsic laziness and self-imposed limitations (Gil-Píriz et al., 2021). Social cognitive theory further asserts that self-efficacy is the pivotal driver of behavioral change (Bandura, 2004). Although smart devices like activity trackers and fitness apps can sustain engagement over short periods (eg, up to 3 months) (Romeo et al., 2019), they cannot substitute for individuals’ inherent exercise volition and intrinsic motivation. Consequently, while smart-sports technologies may modestly reduce the gap, their influence is confined to reinforcing maintenance among those who already exercise, rather than initiating PA behavior (Gabarron et al., 2024). In contrast, psychological cognition constitute the decisive determinants that translate intention into behavior.

Drawing on the important variables identified through causal forest estimation, we find that all important predictors exhibit statistically significant causal associations with the PA I-B gap and that their directional effects align with theoretical expectations (Table 5). Perceived barriers, which yielded the largest average treatment effect (ATE = 0.186), indicate that constraints such as inadequate facility accessibility, lack of exercise methods, weak interpersonal support, diminished interest, and limited time and energy management considerably reduce individuals’ willingness to engage in PA (Lin et al., 2022), thereby widening the gap. The absolute ATEs for self-efficacy and perceived benefits both exceeded 0.10 with negative signs, implying that higher psychological cognition increases the likelihood of translating intention into action. This finding aligns with previous evidence that individuals who recognize the health value of PA and possess high self-efficacy are more likely to adopt effective strategies, invest greater effort, and maintain regular physical activity (Di Maio et al., 2021; Landais et al., 2023). Although the effect size of usage intention toward smart sports tools (ATE = −0.118) was smaller than that of the psychological variables, it was still significantly larger than the ancillary dimensions of social support, personal innovativeness and social influence (all |ATEs| < 0.10). This suggests that, in a mature digital environment, enhancing overall usage intention—by strengthening users’ sense of involvement and motivation—may be a more critical route to narrowing the I-B gap than simply optimizing functional modules. In addition, each one-standard-deviation increase in late-night frequency significantly enlarged the PA I-B gap (ATE = 0.108). Research has proposed that sleep deprivation can lead to depressed mood, fatigue, and impaired decision-making, thereby diminishing individuals’ willingness and capability to participate in exercise (Williams et al., 2024).

Additionally, we generated SHAP dependence plots to examine whether the effects of key features on the PA I-B gap are linear, threshold-based, or non-linear. The results show that perceived barriers, self-efficacy, perceived benefits, stay up late and smart sports tools of personal innovation display monotonic linear relationships with the gap, whereas smart sports tools of usage intention, social support and social influence present more complex,non-linear trajectories. The inverted-U link between usage intention and the PA I-B gap is best interpreted through self determination theory and habit strength. On the left branch, a subset of non-users of smart sports tools is characterized by high autonomy and strong habitual strength (Herrmann and Blackstone, 2020), so technological aids are unnecessary, thus the gap remains small. At the apex of the curve, moderate-intention users often rely on the competence and relatedness supports afforded by the technology for external regulation; when feedback or social incentives are inadequate, intrinsic motivation rapidly erodes (Ryan and Deci, 2000), so the gap widens. On the right branch, most habitual users are exercise enthusiasts with established routines who integrate frequent smart-tool cues into automated behavioral scripts; this transforms external prompts into internalized habits and converges intention with behavior, thereby narrowing the gap. Social support exhibits a clear threshold effect, when emotional support and social interaction from family and friends reach a sufficiently high level, the resulting emotional comfort and peer encouragement markedly reduce psychological barriers and promote sports (Zhao et al., 2024). The gentle U-shaped curve for social influence shows that sedentary adults with a large the gap are more likely to be encouraged by their social circles to adopt smart sports tools for supervised exercise (Sullivan and Lachman, 2016), demonstrating that social influence can provide supplementary facilitation even when cognitive resources are limited. Together, these patterns portray a multidimensional and complex regulatory mechanism in which psychological cognition dominates, while smart tools and social support act in synergy.

To delineate the boundary conditions of the technology acceptance-behavior transformation pathway, we dichotomised perceived barriers and self-efficacy at their respective medians and examined the multidimensional facilitative properties of smart-sports tools across distinct psychological-cognition strata. Across all subgroups, personalized innovation, social support and intention to use exerted significant positive effects. These findings corroborate previous research indicating that tailoring exercise prescriptions to users’ physical condition, training history, goals and preferences (Wu et al., 2020), and reinforcing enjoyment and a sense of belonging through peer interaction and support (Li et al., 2024), is a critical pathway for enhancing exercise maintenance. Ultimately, optimizing these attributes aims to consolidate continued usage intention, thereby enabling smart-sports tools to deliver larger-scale and longer-lasting effects in health management and PA promotion (Migliaccio et al., 2024). Among individuals with low perceived barriers and high self-efficacy, performance expectancy and effort expectancy also exert significant effects, indicating that those with favorable psychological-cognitive profiles are able to fully leverage the diverse functions of smart sports tools. Conversely, only personal innovation, social support and intention to use remained significant among participants with high BAR and low SE, and these effects were amplified in the low-SE group. This suggests that, under conditions of limited psychological-cognition resources, personal innovation, social support and use intention constitute the pivotal dimension for translating intention into behavior, whereas the overall facilitative potential of smart-sports tools is markedly constrained.

Based on the findings of this study, the following recommendations are proposed: Firstly, psychological interventions should be strengthened to reduce perceived barriers and enhance self-efficacy. By improving the allocation of sports resources, promoting scientific exercise methods and fostering a positive sports culture, individuals can be supported in integrating PA into daily life, thereby lowering the barriers to participation. In addition, guiding individuals to set attainable goals and to record their exercise achievements can further elevate self-efficacy and sustain motivation. Secondly, continuously monitor users’ psychological-cognitive states to enable precise alignment of smart-sports tools multifunction. For individuals with elevated perceived barriers or low self-efficacy, priority should be given to activating personal innovation and social-support modules that deliver adaptive exercise prescriptions and create supportive online and offline environments. Such targeted deployment can reduce participation thresholds, boost exercise-specific self-efficacy and facilitate the durable translation of intention into behavior. Third, technology-enablement strategies should be refined to expand the reach of smart-sports tools among psychologically advantaged users. Streamlined interfaces and reduced operational complexity lower technological barriers, while optimized hardware compatibility and environmental adaptation, such as integrated venue booking and weather alerts, enhance the overall exercise experience. Collectively, these measures will encourage sustained engagement, advance population-wide physical activity and contribute to comprehensive health promotion.

## 5 Conclusion

This study innovatively integrates an explainable double-machine-learning framework to examine important predictors of the physical activity intention-behavior gap, grounding the analysis in the Health Belief Model and the Unified Theory of Acceptance and Use of Technology. It introduces a novel relative I-B gap metric to enable cross-population comparisons. Results reveal that psychological cognition, including perceived barriers and self-efficacy, exerts a stronger predictive influence on the gap than smart sports tools, represented by usage intention and social support. Furthermore, the multidimensional attributes of smart sports tools exhibit heterogeneous effects across distinct psychological-cognition subgroups. These findings enrich the theoretical understanding of the I-B gap and provide empirical guidance for designing targeted interventions to bridge the gap and promote physical activity.

## 6 Limitations

While this study provides valuable insights into the PA I-B gap, several limitations should be acknowledged. First, although informed consent was obtained and questionnaires were collected anonymously, and measures such as reverse-worded items and attention checks were employed to partially mitigate response fatigue and consistency motivation, self-reported data remain susceptible to recall and social-desirability biases. Future studies should adopt a multi-method assessment strategy that integrates subjective reports with objective measures (e.g., wearable devices and app log files) to enhance data validity and reliability. Second, the relative I-B gap metric introduced herein is novel and may be subject to unexamined measurement error or limitations; subsequent investigations should employ alternative measurement strategies to corroborate its validity. Third, the cross-sectional design constrains causal inference regarding temporal ordering; longitudinal follow-ups are needed to capture the dynamic evolution of the PA I-B relationship. Moreover, the theoretical framework omits psychological traits beyond self-efficacy and objective environmental factors (e.g., walkability, socio-cultural context). While XGBoost outperformed alternative models, the R^2^ of 0.647 indicates that a substantial proportion of variance in the I-B gap remains unexplained; future studies should incorporate additional variables to improve predictive power. Finally, the sample is exclusively Chinese, which may limit external validity; cross-group validation in culturally diverse and international samples is warranted to enhance generalisability.

## Data Availability

The raw data supporting the conclusions of this article will be made available by the authors, without undue reservation.
